# Supplementary material for: Splice-Junction-Based Mapping of Alternative Isoforms in the Human Proteome
Source: Cell Rep. Author manuscript; Available in PMC 2020 Jan 15. (PMC6961840; doi:10.1016/j.celrep.2019.11.026)
Supplement: 2 [file NIHMS1546469-supplement-2.zip › DF1/PXD006675/RightVentricle/RightVentricle_20_TTN_RVEAEPAEEVTIMEEK.pdf]

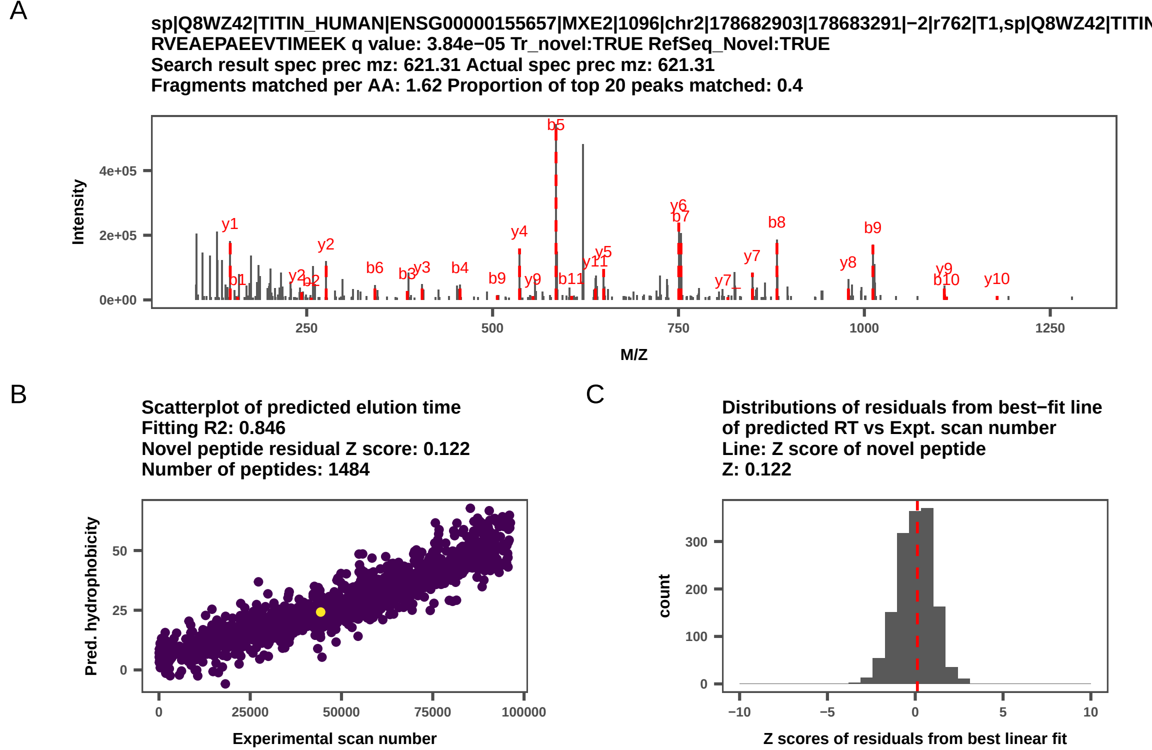
A
sp|Q8WZ42|TITIN_HUMAN|ENSG00000155657|MXE2|1096|chr2|178682903|178683291|−2|r762|T1,sp|Q8WZ42|TITIN
RVEAEPAEEVTIMEEK q value: 3.84e−05 Tr_novel:TRUE RefSeq_Novel:TRUE
Search result spec prec mz: 621.31 Actual spec prec mz: 621.31
Fragments matched per AA: 1.62 Proportion of top 20 peaks matched: 0.4
Intensity
4e+05
2e+05
0e+00
b5
y6
b7
b8
b9
y1
y2
y4
y5
y11
y7
y8
y9
b10
y10
b1
y2
b2
b6
b3
y3
b4
b9
y9
b11
y7
250
500
750
1000
1250
M/Z
B
Scatterplot of predicted elution time
Fitting R2: 0.846
Novel peptide residual Z score: 0.122
Number of peptides: 1484
Pred. hydrophobicity
50
25
0
0
25000
50000
75000
100000
Experimental scan number
C
Distributions of residuals from best−fit line
of predicted RT vs Expt. scan number
Line: Z score of novel peptide
Z: 0.122
count
300
200
100
0
−10
−5
0
5
10
Z scores of residuals from best linear fit
